# Lipid droplets and perilipins in canine osteosarcoma. Investigations on tumor tissue, 2D and 3D cell culture models

**DOI:** 10.1007/s11259-022-09975-8

**Published:** 2022-07-14

**Authors:** N. Leitner, J. Hlavatý, R. Ertl, S. Gabner, A. Fuchs-Baumgartinger, Ingrid Walter

**Affiliations:** 1 Institute of Morphology, Working Group Histology, University of Veterinary Medicine, Veterinaerplatz 1, A-1210 Vienna, Austria; 2 VetCore Facility for Research, University of Veterinary Medicine, Veterinaerplatz 1, A-1210 Vienna, Austria; 3 Institute of Pathology, University of Veterinary Medicine, Veterinaerplatz 1, A-1210 Vienna, Austria

**Keywords:** Canine osteosarcoma, Lipid droplets, Perilipins, 3D spheroid model

## Abstract

Lipid droplets were identified as important players in biological processes of various tumor types. With emphasis on lipid droplet-coating proteins (perilipins, PLINs), this study intended to shed light on the presence and formation of lipid droplets in canine osteosarcoma. For this purpose, canine osteosarcoma tissue samples (*n* = 11) were analyzed via immunohistochemistry and electron microscopy for lipid droplets and lipid droplet-coating proteins (PLINs). Additionally, we used the canine osteosarcoma cell lines D-17 and COS4288 in 2D monolayer and 3D spheroid (cultivated for 7, 14, and 21 days) *in vitro* models, and further analyzed the samples by means of histochemistry, immunofluorescence, molecular biological techniques (RT-qPCR, Western Blot) and electron microscopical imaging. Lipid droplets, PLIN2, and PLIN3 were detected in osteosarcoma tissue samples as well as in 2D and 3D cultivated D-17 and COS4288 cells. In spheroids, specific distribution patterns of lipid droplets and perilipins were identified, taking into consideration cell line specific zonal apportionment. Upon external lipid supplementation (oleic acid), a rise of lipid droplet amount accompanied with an increase of PLIN2 expression was observed. Detailed electron microscopical analyzes revealed that lipid droplet sizes in tumor tissue were comparable to that of 3D spheroid models. Moreover, the biggest lipid droplets were found in the central zone of the spheroids at all sampling time-points, reaching their maximum size at 21 days. Thus, the 3D spheroids can be considered as a relevant *in vitro* model for further studies focusing on lipid droplets biology and function in osteosarcoma.

**Supplementary Information:**

The online version contains supplementary material available at 10.1007/s11259-022-09975-8.

## Introduction

Lipid droplets (LDs) in human osteosarcoma (OS) were discovered decades ago within the context of ultrastructural studies (Garbe et al. 1981), but they have not drawn much attention at that point in time. Recently, the recognition and importance of LDs has significantly risen in normal cells as well as in tumor cells. Lipids and the lipid metabolism of tumor cells is now in focus of tumor research, however data regarding the function of lipids in OS is lacking. The canine OS resembles in many characteristics the human OS (Fenger et al. 2014; Fan and Khanna 2015); therefore, the dog is an accepted model for the human disease and results obtained by experimental approaches are significant for both species (Simpson et al. 2017). Single studies undertaken in this field raised important issues, for example, an association of lipids with metastatic behavior of human OS cells (Roy et al. 2019). Meanwhile it is well accepted that LDs are not only storage compartments but also act as complex organelles and signaling platforms; their functions in tumor pathogenesis, growth and progression warrant further investigation.

The importance of LDs has been ascertained in several tumor types in humans such as breast cancer (Vidavsky et al. 2019), colorectal cancer (Tirinato et al. 2015), glioblastoma (Liu et al. 2021), prostate cancer (Fontaine et al. 2021). Only occasional observations published as case reports are available in dogs (Hayden et al. 1993). LDs in tumor cells or in adipose tissue adjacent to tumor cells are primarily seen as energy sources, aiding tumor growth. Taking glioma as an example, lipolysis of LDs was found to fuel tumor progression in humans (Liu et al. 2021). Growth and invasiveness of human prostate carcinoma cells are driven by lipid uptake and *de novo* lipogenesis and if inhibited, tumor cell growth was suppressed (Laurent et al. 2019). Interestingly, the role of LDs in humans varies depending on cancer type (Shang et al. 2020), and there is conflicting data regarding the prognostic value of LDs. Several human studies have identified enhanced LD content of tumor cells (breast cancer, prostate cancer) as a marker of aggressive tumor phenotypes (reviewed in Li et al. 2020). The metastatic potential of cancer cells in several tumor types was positively correlated with intracellular lipid storage (Li et al. 2020). On the other hand, it has been reported that epithelial-mesenchymal transition-derived human breast cancer cells can be directed to differentiate into post-mitotic adipocytes (Ishay-Ronen et al. 2019). In this case, LD accumulation resulted in a tumor repressive process.

Most LDs comprise a hydrophobic core of triacylglycerols (TAG) and/or sterol esters (SE) surrounded by a monolayer of phospholipids (Yang et al. 2012). An integral part of the outside of LDs are so-called lipid droplet coating proteins, among which the perilipins (PLINs) play an important role in LD formation and stability (Itabe et al. 2017; Zhang et al. 2018). In mammals, the PLIN family consists of five members (PLIN1-5) with different functions: PLIN1 and PLIN2 are clearly associated with LDs, PLIN3 and PLIN5 are LD-associated but also cytoplasmic, and finally, PLIN4 is predominantly membranous in skeletal muscle (Itabe et al. 2017; Pourteymour et al. 2015). PLIN1 plays an important role in LD stabilization, formation and triglyceride metabolism, as well as in controlling lipolysis in adipocytes (reviewed in Itabe et al. 2017; Sztalryd and Brasaemle 2017). Apart from LD formation and stabilization, PLIN2 can protect triglycerides from lipolysis (Itabe et al. 2017). PLIN3 induces the production of prostaglandin E2 in neutrophils (Nose et al. 2013) and was reported to be enhanced in exercising human muscle cells (Covington et al. 2014). PLIN4 plays a role in the formation of LDs in adipocytes (Itabe et al. 2017). PLIN5 is predominantly found in oxidative tissues (i.e. skeletal and heart muscle). This protein mediates a connection between LD and mitochondria and presumably displays a cytoprotective effect via decreasing fatty acid toxicity (reviewed in Kimmel and Sztalryd 2014). PLINs have been shown as suitable markers for LDs in human tumors (Zhang et al. 2018, 2020, 2021). In breast cancer, high expression of PLIN1 predicted a longer overall patient survival, while overexpression of PLIN2 indicated poor overall patient survival (Zhang et al. 2021). Determining the respective PLIN proteins aided the differentiation of tumor types in liposarcomas (Zhang et al. 2020). A risk score model based on lipid metabolism related genes showed potential for survival prediction in human OS (Quian et al. 2021). Therefore, understanding the osteosarcoma lipid metabolism might be of high diagnostic, prognostic, and therapeutic value.

Cancer cells subjected to hypoxia or nutrient starvation have an outstanding ability to synthesize fatty acids and often show an increased accumulation of LDs in human (Cabodevilla et al. 2013; Schlaepfer et al. 2015) and rat (Cabodevilla et al. 2013). During growth of microtumors and establishment of metastases, hypoxia often occurs due to lack of vascularization (Lunt et al. 2009). Therefore, we intended to mimic tumor development with a spheroid tumor model of canine OS cells. Spheroids are known to represent a microtumor with an outer zone with full access to nutrients and oxygen whereas the central zone is depleted from resources (Lin and Chang 2008; Gebhard et al. 2015). Not only an oversupply of external fat supports LD accumulation in tumor cells, also starvation and other stress factors lead to a comparable outcome. Differences in expression of lipid processing genes and total lipid content between diverse cell populations in human glioblastoma organoids with lipid enrichment in hypoxic organoid cores were reported (Shakya et al. 2021). This correlation of LDs and hypoxia/stress needs attention as previous studies demonstrated that oxidative stress promotes invasiveness of U-2OS osteosarcoma cells *in vitro* (Shin et al. 2016). Therefore, the association between lack of regular vascularization, hypoxia, stress, accumulation of LDs, and promotion of invasion and metastatic behavior in tumors warrants further investigations *in vitro* and *in vivo*.

The first goal of our study was to analyze the incidence of LDs (via p-phenylenediamine staining and assessing the LD coating PLIN1, PLIN2, PLIN3) and size of LDs (via transmission electron microscopy) in canine OS tumor specimens derived from patients after surgery or necropsy. The second aim was to investigate LD occurrence, size, amount, and formation in 2D and 3D canine osteosarcoma cell culture models using the commercially available D-17 cell line and COS4288, a canine patient derived osteosarcoma cell line established in our laboratory. We analyzed LDs and PLINs by means of histochemistry, immunofluorescence, Western Blotting, RT-qPCR, and transmission electron microscopy. Cholesterol (Chol) and TAG content in the 2D and 3D cell cultures were analyzed quantitatively. In addition, dynamics of LD formation provoked by external lipid stimulation was investigated. These experiments help to gain further understanding about the lipid metabolism in OS cells and indicate the applicability of the spheroid culture for OS lipid studies.

## Material and methods

### Tissue samples

Tissue samples from canine OS patients (*n* = 11) were acquired by the VetBioBank/VetCore archive at the University of Veterinary Medicine Vienna. Inclusion criteria for the study were canine patients diagnosed with OS of the appendicular skeleton. The sampling procedure followed the standard operation protocol from VetBioBank (Walter et al. 2020). Information about breed, age, gender and specific tumor location are part of the above-mentioned standard operation procedure. All tissue samples were obtained from chemotherapy naïve patients via surgery or necropsy according to ethical rules and legal standards from the University of Veterinary Medicine Vienna. Formalin-fixed (4% neutral buffered formaldehyde, Liquid Production GmbH, Flintsach am Inn, Germany), paraffin-embedded (FFPE) tissue samples were cut in 3 μm sections and stained with hematoxylin and eosin (H&E) according to Romeis (1989) for general analyses. H&E stained sections of OS samples were classified and graded histopathologically by an experienced pathologist, considering the guidelines of Loukopoulos and Robinson (2007) (Table 1).

**Table 1:** Canine osteosarcoma samples used in the study

Number	Breed	Age (years)	Gender	Medical intervention	Tumor location	Tumor type	Tumor grade
1	Crossbreed	10.5	Male	Amputation	Femur	Osteoblastic OS	I
2	German short hair	1.5	Female	Amputation	Tibia	Chondroblastic OS	III
3	Rottweiler	9	Male	Amputation	Tibia	Osteoblastic OS	II
4	Crossbreed	9	Male	Amputation	Metatarsus	Osteoblastic OS	I
5	Rottweiler	9	Female	Amputation	Humerus	Osteoblastic OS	II
6	Crossbreed	10	Male	Amputation	Humerus	Osteoblastic OS	III
7°	Landseer	5	Male	Euthanasia	Humerus	Osteoblastic OS	II
8	Bullmastiff	10	Male	Euthanasia	Humerus	Chondroblastic OS	II
9	Crossbreed	1	Female	Amputation	Tibia	Osteoblastic OS	I
10*°	Labrador	8.5	Female	Amputation	Femur	Osteoblastic OS	III
11°	Australian shepherd	10	Male	Scapulectomy	Scapula	Chondroblastic OS	III

^*^ cell culture, ° transmission electron microscopy

### Cell culture under standard condition

Canine D-17 osteosarcoma cells were obtained by ATCC (Manassas, VA, USA; cat. nr. CCL-183; RRID:CVCL_1916) and cultured in MEM (PAN Biotech, Aidenbach, Germany) supplemented with 10% fetal calf serum, 1% antibiotic–antimycotic solution (both Sigma-Aldrich, St. Louis, MO, USA) and 1% L-glutamine (Biowest, Riverside, MO, USA). COS4288 cells were isolated from an osteoblastic canine OS tissue sample (Nr. 10, Table 1) obtained by VetBioBank/VetCore at the University of Veterinary Medicine Vienna. Briefly, tumor tissue sample was washed in PBS (Sigma-Aldrich, St. Louis, MO, USA) supplemented with 2% antibiotic–antimycotic solution and dissected into small pieces. Tissue pieces (1-2mm^3^) were placed in DMEM HG (Sigma-Aldrich, St. Louis, MO, USA), supplemented with 10% fetal calf serum, 1% antibiotic–antimycotic solution and 1% L-glutamine in an incubator at 37 °C and 5% CO_2_. After cell outgrowth was provable, tissue pieces were removed and adherent cells were further cultivated and characterized as of mesenchymal origin, with hallmarks of malignant transformation (unlimited proliferation capacity and anchorage-independent cell growth) and as of OS origin (see Supplementary data). Both, D-17 and COS4288 cells were regularly passaged 2–3 times a week using trypsin solution (Trypsin–EDTA, BioWest) with a usual split ratio of 1:5.

For 3D spheroid cell culture, both cell lines were seeded on a 96-well low attachment plate (96-well BRANDplates® U inert Grade™ clear, Brand GmbH und Co KG, Wertheim, Germany) in a concentration of 2 × 10^4^ cells/well for D-17 and 4 × 10^4^ cells/well for COS4288 cells and cultivated for 7, 14 and 21 days. Half of the respective medium volume (from 200 µl total volume) was changed every second day. The produced spheroids were used in experiments as described below.

Described culture media are referred to as “standard conditions” (normal medium, NM).

### Cell culture with lipid supplementation

For lipid treatment experiments, cells grown as a monolayer were treated with oleic acid (OA) (Sigma Aldrich, final conc. 56.5 µg/ml), cholesterol (Sigma Aldrich, final conc. 50 µg/ml) or a combination thereof (56.5 µg/ml OA + 50 µg/ml Chol) for 24 h. Alternatively, four days old spheroids were treated with 56.5 µg/ml OA or 50 µg/ml Chol for additional ten days; 100 µl out of 200 µl medium per well was changed every second day. As both OA and Chol were dissolved in ethanol, cells grown in medium containing ethanol (final conc. 0.5%) were used as a solvent control. Cells were further processed according to the requirements for respective analyses described below. Unless otherwise indicated, all cell culture experiments were performed in three independent replicates.

### Oil Red O staining of cell cultures

Oil Red O staining was performed to demonstrate LDs in D-17 and COS4288 cells grown as 2D monolayers as well as in 3D spheroids cultured for 7, 14, 21 days. For monolayer experiments, cells were grown on glass cover slips in a 24-well format (seeding concentration 5 × 10^4^ cells per well). Afterwards, they were washed with PBS, fixed in 4% neutral buffered formaldehyde for 10 min at room temperature and stained with Oil Red O as described in Romeis (1989). Nuclei were counterstained with hematoxylin. For microscopic analysis, coverslips were fixed to glass slides upside down with Aquatex (Merck, Darmstadt, Germany). To analyze cells grown in 3D, spheroids were collected out of the 96-well plate, washed with PBS, allowed to sediment, embedded in OCT Compound (ThermoScientific, Runcorn, UK) and frozen at -20 °C. Cryosections (5 µm) were fixed in 4% neutral buffered formaldehyde for 10 min at room temperature followed by Oil Red O staining procedure as mentioned above.

For colorimetric quantification of Oil Red O staining, D-17 and COS4288 cells were grown directly in wells of a 24-well plate (5 × 10^4^ cells/well) as monolayers for 24 h. Afterwards, cells were treated with lipid supplements for additional 24 h and Oil Red O stained as described above, omitting the nuclear counterstaining step. The Oil Red O signal was spectrophotometrically quantified as described previously by Kraus et al. (2016). Briefly, Oil Red O stained cells were air dried and incubated with 500 µl/well isopropanol for 15 min at room temperature to dissolve the Oil Red O stain. Afterwards, 200 µl of the respective solution was applied in duplicate to a flat and clear 96-well plate (Greiner BioOne, Kremsmünster, Austria). Absorbance was read at 510 nm using a Microplate Reader (Infinite M200 *Pro*, Tecan, Grödig, Austria).

### Immunofluorescent detection of PLINs

For immunofluorescent staining, tumor tissues (*n* = 11), D-17 and COS4288 monolayer and spheroid samples grown under standard conditions (total spheroid cultivation time 7, 14 and 21 days) or with lipid supplements (total spheroid cultivation time 14 days) were used. Cell culture samples for immunofluorescence analyses were grown in 2D on chamber slides (Lab-Tek® II Chamber Slide System, ThermoFisher Scientific, Waltham, MA, USA) and fixed in 4% neutral buffered formaldehyde prior to further analyses. D-17 and COS4288 spheroid samples were fixed in 4% neutral buffered formaldehyde, and overlaid with Histogel™ (Richard-Allan Scientific, Walldorf, Germany) before paraffin embedding. Subsequently, 3D cell culture samples and tumor tissues were cut in 3 µm sections, deparaffinized in xylene and rehydrated. Endogenous peroxidases were blocked by incubating the sections in 3% H_2_O_2 _(Carl Roth) for one hour at room temperature. Antigen retrieval was performed in 0.01 M citrate buffer (pH 6.0) for 30 min at 85 °C for PLIN1 and PLIN2. For PLIN3 immunofluorescence, antigen retrieval was not required. After a washing step in PBS (pH7.4), samples were incubated in 10% normal goat serum (Sigma Aldrich) for one hour in a humidified chamber to prevent unspecific antibody binding. Incubation with the respective primary antibody was performed over night at 4 °C, followed by secondary antibody incubation for one hour at room temperature (Table 2). Signal amplification with Tyramide solution was utilised (AlexaFluor 568 Tyramide Reagent, Invitrogen, Waltham, MA, USA). Nuclei were visualized using DAPI (4′,6-diamidino-2-phenyl-indol-dihydrochloride, 0.2 mg/ml, Sigma Aldrich) in PBS for 3 min. Finally, sections were mounted with AquaPoly Mount (Polysciences, Warrington, PA, USA). The fluorescence signal was evaluated using a confocal laser scanning microscope (Zeiss CLSM 880 Airyscan, Oberkochen, Germany) and the program ZEN Black edition (Zeiss).

**Table 2:** Antibodies used for immunofluorescence and Western Blot detection

Antibody name	Method	Antibody typ	Source	Catalogue nr	Dilution
anti-PLIN1 (anti-PLIN1 rabbit polyclonal antibody)	IF	primary	Abcam Cambridge, UK	ab3526 RRID:AB_2167274	1:10 000
anti-PLIN2 (ADRP (Perilipin 2) (AA5-27 AP125) mouse monoclonal antibody)	IF	primary	AntibodiesOnline Aachen, Germany	ABIN112185 RRID:AB_2915956	1:500
anti-PLIN3 (TIP47 (F-10) mouse monoclonal antibody)	IF	primary	Santa Cruz Biotechnologies Santa Cruz, CA, USA	sc-390968 RRID:AB_2915955	1:5 000
BrightVision, 1 step detection system goat anti- mouse HRP	IF	secondary	ImmunoLogic Duiven, Netherlands	DPVM110HRP RRID:AB_2915957	RTU
BrightVision, 1 step detection system goat anti- rabbit HRP	IF	secondary	ImmunoLogic Duiven, Netherlands	DPVR110HRP RRID:AB_2915958	RTU
anti-PLIN1 (anti-PLIN1 rabbit polyclonal antibody)	WB	primary	Abcam Cambridge, UK	ab3526 RRID:AB_2167274	1:500
anti-PLIN2 (ADRP/Perilipin 2 rabbit polyclonal antibody)	WB	primary	Proteintech Rosemont, IL, USA	15,294–1-AP RRID:AB_2878122	1:500
anti-PLIN3 (TIP47 (F-10) mouse monoclonal antibody)	WB	primary	Santa Cruz Biotechnologies Santa Cruz, CA, USA	sc-390968 RRID:AB_2915955	1:500
anti-GAPDH (GAPDH [GT239] mouse monoclonal antibody)	WB	primary	GeneTex Irvine, CA, USA	GTX627408 RRID:AB_11174761	1:5 000
anti-α-tubulin (anti-α-tubulin rabbit polyclonal antibody)	WB	primary	Abcam Cambridge, UK	ab4074 RRID:AB_2288001	1:1 000
Amersham ECL Rabbit IgG, HRP-linked whole Ab (donkey)	WB	secondary	GE Healthcare Chicago, IL, USA	NA934 RRID:AB_772206	1:5 000
Amersham ECL Mouse IgG, HRP-linked whole Ab (sheep)	WB	secondary	GE Healthcare Chicago, IL, USA	NA931 RRID:AB_772210	1:5 000

IF – immunofluorescence; WB – Western Blot; RTU – ready to use

Positive and negative controls were included in every staining process. Sections of canine adipose tissue (PLIN1) and canine adrenal gland (PLIN2 and PLIN3) served as positive controls. To monitor unspecific binding of the detection system, negative controls where the primary antibody was substituted with PBS, were used.

### Western Blot for PLINs

For cell lysate preparation, monolayer cells (D-17 and COS4288) were washed with PBS, scraped off the cell culture flask, centrifuged, and the resulting cell pellet was stored at -80 °C. Spheroids (D-17 and COS4288) grown under standard condition (cultivation time 7, 14 and 21 days) or with lipid supplements (cultivation time 14 days) were collected from the 96-well plate, washed with PBS, allowed to sediment and stored as a dry pellet at -80 °C until further processing.

Western Blot samples were lysed in RIPA buffer (50 mM Tris–HCl pH 7.4, 500 mM NaCl, 0.5% sodium deoxycholate (all Carl Roth GmbH, Karlsruhe, Germany), 1% Nonidet P-40 (Igepal, Sigma Aldrich), 0.1% sodium dodecyl sulfate (Serva, Mannheim, Germany)) supplemented with 1% protease and phosphatase inhibitors (Protease Inhibitor Cocktail and Phosphatase Inhibitor Cocktail 3; both Sigma Aldrich). Mechanic disintegration was used to promote lysis. Protein concentration was measured using DC™ Protein Assay (BioRad, Hercules, CA, USA) according to the manufacturer’s instructions. Twenty microgram of protein extract per sample were loaded per lane.

Cell lysates were separated on a 10% polyacrylamide gel using the BioRad Mini Protean Tetra System (BioRad) and transferred to a PVDF Membrane (GE Healthcare, Chicago, IL, USA). To prevent unspecific antibody binding, blocking was performed using Western Blot Blocking Reagent (Roche, Basel, Switzerland) diluted in TBST (1:10) for 2 h at room temperature. PLIN1, PLIN2 and PLIN3 primary antibodies were incubated at 4 °C over night, followed by an intensive washing step (5 × 8 min in TBST). Afterwards, membranes were incubated with the respective secondary antibody for 30 min at room temperature and washed again (4 × 8 min in TBST, 1 × 8 min in TBS). All antibodies were diluted in Western Blot Blocking Reagent (Roche)/TBST (1:10). The signal was visualized using ECL Western Blot Detection Reagents (GE Healthcare, Chicago, IL, USA) and BioRad ChemiDoc Image System with Image Lab Software (both BioRad). Antibodies used are listed in Table 2.

### Quantitative real-time PCR

D-17 and COS4288 cells (grown as monolayer and spheroids under standard condition, total spheroid cultivation time 7, 14 and 21 days) were harvested, lysed in TRI Reagent (Zymo Research, Irvine, CA, USA) and stored at -80 °C until further processing. RNA extraction and DNase I treatment were done with the Direct-zol RNA Miniprep Kit (Zymo Research) according to the manufacturer’s instructions. Cells cultured as spheroids were mechanically homogenised on a MagNA Lyser instrument (Roche, Rotkreuz, Switzerland) using 1.4 mm ceramic beads (Qiagen, Hilden, Germany) at 6500 rpm for 30 s prior to extraction. Reverse transcription (RT) was performed with the High-Capacity cDNA Reverse Transcription Kit (ThermoFisher Scientific). No-RT controls (without RT enzyme) were included for each sample to monitor the amplification of contaminating DNA. Primers for RT-qPCR (Table 3) were designed with the PrimerQuest primer design tool (https://eu.idtdna.com/PrimerQuest/Home/Index; Integrated DNA Technologies, Coralville, IA, USA) or taken from literature (Gabriel et al. 2016). The qPCR was done in 20 µl reaction volumes including 1 × HOT FIREPol EvaGreen qPCR Mix Plus ROX (Solis BioDyne, Tartu, Estonia), 200 nM of each primer and 30 ng cDNA. All samples were analysed in duplicates on a AriaMx Real-time PCR System (Agilent, Santa Clara, CA, USA) with following temperature profile: 95 °C for 12 min, 40 cycles of 95 °C for 15 s and 60 °C for 1 min, followed by a melting curve step (60–95 °C). Four potential reference genes (RGs): OAZ1, RPL8, RPL27 and RPL32 were included for normalization. The RG expression stability was assessed with the RefFinder tool (Xie et al. 2012). The two most stably expressed genes (OAZ1 and RPL27) were selected for normalization. Target gene Ct values were normalized to the mean of the selected RGs and relative fold changes were calculated with the comparative 2^−ΔΔCT^ method (Livak and Schmittgen 2001).

**Table 3:** Primer for RT-qPCR

Gene symbol	Gene name	NCBI accession number	Oligo sequence (5 ‘—3 ‘)	Amplicon length (bp)	PCR efficiency (%)	R^2^ value	Reference
PLIN1	Perilipin 1	XM_038661340.1	F: GTACCCTCCTGAGAAGATTG R: GGGCACACTGATGCTATT	85	98	0.993	-
PLIN2	Perilipin 2	XM_005626663.3, XM_003639380.4	F: AATTTGCCAGAAAGAATGTGCAT R: TCCACCCAGGAGAGGTAGAACTT	79	99	0.991	-
PLIN3	Perilipin 3	XM_038429023.1, XM_038429022.1	F: GGGTCAGGAGAAACTACAC R: GTCTCCACCTCTGGTTTG	93	97	0.995	-
OAZ1	Ornithine decarboxylase antizyme 1	NM_001127234.1	F: CTGCTGTAGTAACCTGGGTC R: ACATTCAGCCGATTATCAGAGTA	145	97	0.994	Gabriel et al. 2016
RPL27	Ribosomal protein L27	NM_001003102.2	F: ACTACAATCACCTCATGCCC R: CTTGTACCTCTCCTCGAACTTG	143	94	0.998	Gabriel et al. 2016
RPL32	Ribosomal protein L32	NM_001252169.1	F: TGGCCATCAGAGTCACCAATC R: GACGCGCACATAAGCTGTTTAT	74	94	0.998	-
RPL8	Ribosomal protein L8	XM_853403.4	F: TCTTCCGCCAACAGAGCC R: CTTTGCCTTGTACTTGTGGTAAGC	102	94	0.995	-

R^2^: correlation coefficient of standard curve; F and R: forward and reverse primer

### Colorimetric quantification of cholesterol and triacylglycerides in cell cultures

For colorimetric quantification of Chol and TAG, D-17 monolayer cells (1 × 10^6^ for the Chol quantification assay and 1 × 10^7^ for the TAG quantification assay) and spheroids (96 spheroids for the Chol quantification assay and 480 spheroids for the TAG quantification assay) cultured under standard conditions (cultivation time 7, 14 and 21 days) were used. A Cholesterol/Cholesteryl Ester Quantitation Assay Kit (Abcam, Cambridge, UK) and Triglyceride Quantification Assay Kit (Abcam, Cambridge, UK) were used according to the manufacturer’s instructions with slight modification concerning solvent vaporization in the cholesterol kit: sample incubation for 3 h at 50 °C on a heating block was utilized instead of 30 min vacuum centrifugation.

Absorbance was read at 570 nm in flat and clear 96-well plates (Greiner BioOne) using a Microplate Reader (Infinite M200 *Pro,* Tecan). The levels of TAG, total cholesterol and free cholesterol were measured, and the amount of cholesterol esters was calculated according to the kit instructions.

### Transmission Electron Microscopy (TEM)

To further analyze LD in osteosarcoma tumor tissue (*n* = 3, Table 1) as well as in D-17 and COS4288 cells cultivated as monolayer or spheroids (under standard conditions, total spheroid cultivation time 7, 14, 21 days) were used. All samples were fixed in 3% buffered glutaraldehyde (pH 7.4, Merck). Cell culture specimens were pre-embedded in 1.5% agar. After being washed in 0.1 M Soerensen buffer (pH 7.4), the samples were postfixed for 2 h at room temperature in 1% osmium tetroxide (Electron Microscopy Sciences, Hatfield, PA, USA). This was followed by dehydration in an ethanol series along with an increasing series of propylene oxide (Sigma Aldrich) before embedding and polymerization in epoxy resin (Serva, Mannheim, Germany) for 48 h at 60 °C.

Ultrathin sections (70 nm) were cut for transmission electron microscopic evaluation and contrasted in methanolic uranyl acetate (Fluka Chemie AG, Buchs, Switzerland) and alkaline lead citrate (Merck). For imaging, a transmission electron microscope (EM 900, Zeiss, Oberkochen, Germany) and ImageSP Professional software (SYSPROG, TRS, Moorenweis, Germany) were used.

To measure the size of LDs in monolayer and spheroids of D-17 and COS4288 cells (timepoints: 7, 14, 21 days) as well as in the original tumor sample from which COS4288 cells were isolated (Nr. 10, Table 1), microphotographs were randomly taken from the analyzed subjects, taking into consideration the different zones in spheroids. Zonal analysis of LDs by TEM was performed as follows: In D-17 spheroids, the outer zone images included the edge of the spheroid, pictures representing the central zone included the necrotic area and images of the intermediate zone were taken outwards adjacent to the necrotic center.

In COS4288 spheroids, images representing the outer zone (where the cells were densely packed) included the edge of the spheroids; the central zone was represented by loosely arranged cells. At least 50 LDs (in spheroids per zone and timepoint) were measured in two dimensions with ImageSP Viewer (SYSPROG, TRS). The mean values were taken for further statistical analysis.

### P-phenylenediamine (PPD) staining

Staining with PPD was performed to visualize lipid droplets in OS tissues and 3D spheroids postfixed in osmium tetroxide and then embedded in epoxy resin. Semi-thin sections (0.8 µm) from tumor tissue and 3D spheroids (cultivated under standard conditions, timepoints: 7, 14, 21 days) of both cell lines were stained in a 1% p-phenylenediamine (Sigma Aldrich) ethanol solution for 5 min. After a washing step in distilled water, sections were mounted with Aquatex (Merck).

### Statistics

Statistical analyses (unpaired t-test with Welch’s correction) were conducted with GraphPad Prism 8.4.3 (GraphPad Software, San Diego, CA, USA) in quantitative analysis of Oil Red O staining; WB, PCR, lipid assays and electron microscopic measurements. A *p*-value < 0.05 was considered as statistically significant.

## Results

### Lipid droplets in osteosarcoma tissue

Out of the 11 H&E stained tissue samples (Table 1), 8 were diagnosed as osteoblastic OS, with the remaining 3 being diagnosed as chondroblastic OS. Among the samples, 3 were scored as grade I, 4 scored as grade II, and 4 scored as grade III. All dogs included in this study were neutered, with the male:female ratio of 7:4. The age varied between 1 and 10.5 years, with a mean value of 7.6 years.

**Fig. 1:**
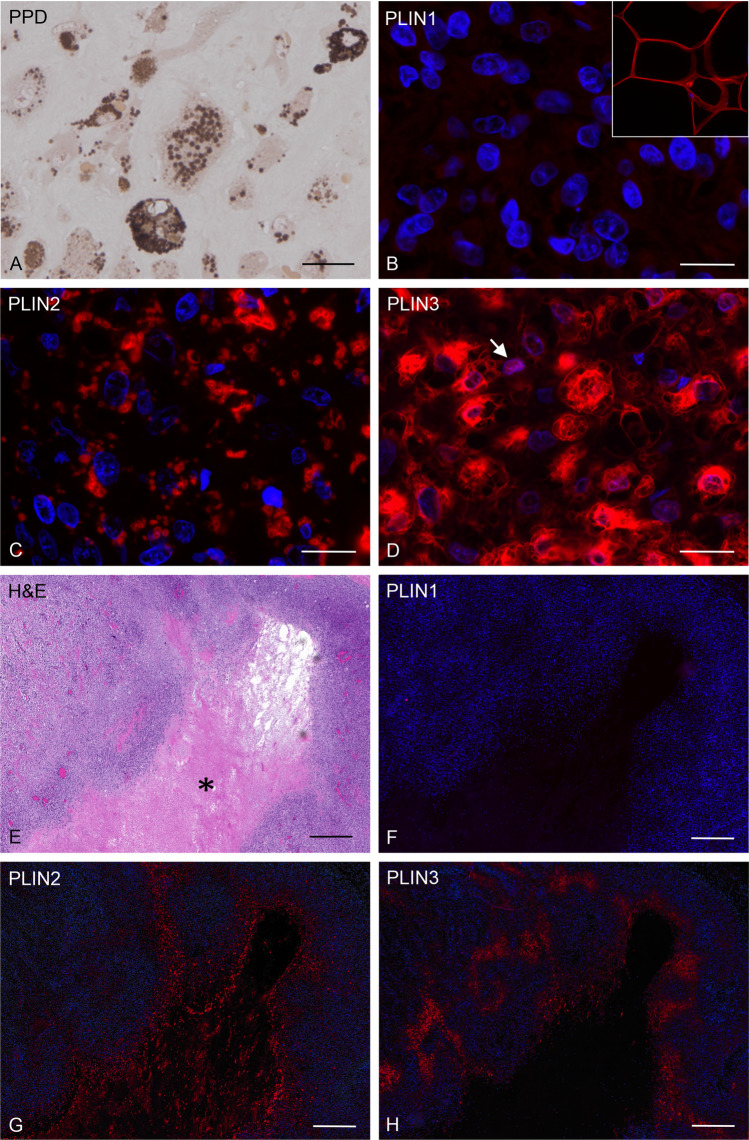
Canine osteosarcoma tissue samples originating from COS4288 primary tumor. PPD staining (brown signal) visualized lipid droplets in a semithin section (A). Signals were heterogeneously distributed in the cells. PLIN1 revealed no signal in tumor cells (B). Insert shows positive staining (red) in canine adipose tissue used as positive control. Immunofluorescence detection of PLIN2 (red) revealed its localization to lipid droplet membrane (C). Immunofluorescence detection of PLIN3 (red) was localized in the lipid droplet membranes as well as in the cytoplasm, with occasional signal in the nucleus (arrow; D). H&E staining of an osteoblastic osteosarcoma with a prominent necrotic area (asterisk, E). Serial sections showing corresponding tumor areas immunostained for PLIN1 (F), PLIN2 (G), and PLIN3 (H). No PLIN1 signal was present in this tumor area. Clear differences were seen between PLIN2 and PLIN3 staining pattern. PLIN2 signals were observed within and adjacent to the necrotic area but were also prominent in other regions. PLIN3 was observed predominantly in necrosis surrounding areas. Nuclei were counterstained with DAPI Scale bars 200 µm (A-D) and 500 µm (E–H)

A substantial amount of LDs was demonstrated by PPD staining on semithin sections of resin-embedded specimens, where the lipids themselves were preserved via osmium tetroxide in contrast to paraffin embedding techniques (Fig. 1A). Positive PLIN1 fluorescence signals were restricted to adipocytes that were occasionally present in tumor tissue surroundings. As for the cellular localization, PLIN1 signal was bordering giant lipid droplets (Fig. 1B). PLIN2 immunofluorescence clearly visualized LD contour, showing distinct circles corresponding to LD membranes on paraffin sections and revealed a considerable amount of irregularly distributed LD containing cells (Fig. 1C). PLIN2 positive cells were present in a highly variable amount in all tumor samples. Immunoreactivity for PLIN3 was also heterogeneously distributed within the tumors. The subcellular distribution of the PLIN3 signal was distinct from PLIN2, showing a more cytoplasmic pattern, additional LD membrane corresponding circles, and occasional signals in the nuclei (Fig. 1D). Serial sections of the osteoblastic primary tumor of COS4288 indicated vital and necrotic areas (Fig. 1E). Immunofluorescence for PLIN1 showed no signal within the tumor (Fig. 1F), whereas PLIN2 positive cells were seen adjacent and within the necrotic region (Fig. 1G) and PLIN3 was restricted to the necrosis surrounding area (Fig. 1H). Both were also present in vital areas in irregular distribution (Fig. 1G, H).

### Lipid droplets in osteosarcoma cells *in vitro*

**Fig. 2:**
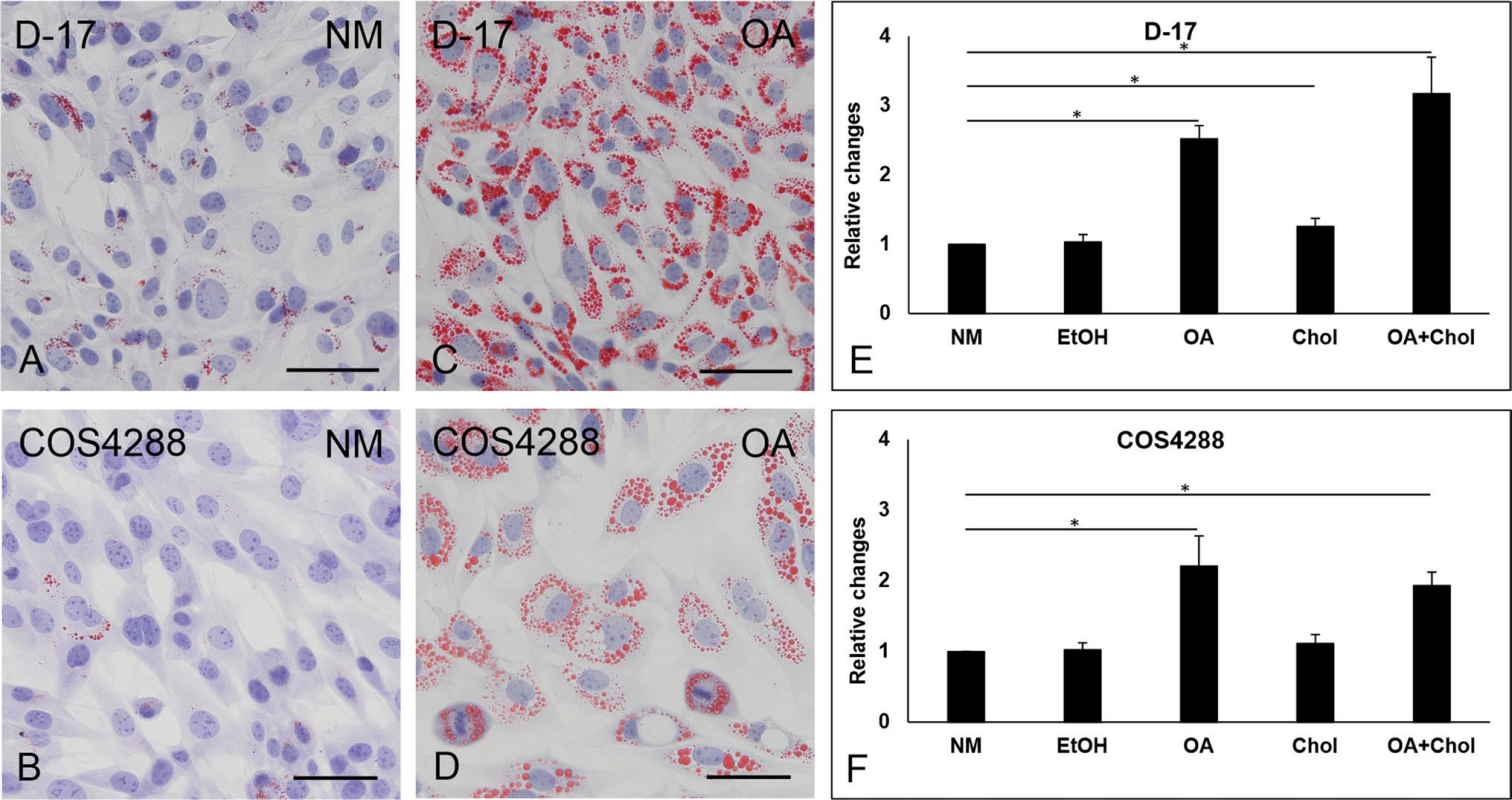
Oil Red O staining of lipid droplets and colorimetric quantification of the Oil Red O staining in canine osteosarcoma cell lines cultivated in a monolayer. A small number of lipid droplets (red signal) was detected in D-17 (A) and COS4288 (C) cells cultivated under standard conditions without lipid supplementation. A dramatic increase in the amount and size of lipid droplets was observed after 24 h treatment with 56.5 µg/ml of oleic acid in both D-17 (B) and COS4288 (D) cells. Scale bar 500 µm. For the colorimetric quantification of the Oil Red O staining in D-17 (E) and COS4288 (F) cells, staining intensity obtained in cells without lipid supplementation (NM) was arbitrary set to 1, other bars represent values relative to these. Treatment with oleic acid (alone or in combination with cholesterol) resulted in an increase of staining intensity. NM – normal medium/no supplementation, EtOH – 0.5% ethanol (solvent control), OA – 56.5 µg/ml oleic acid, Chol – 50 µg/ml cholesterol, OA + Chol – 56.5 µg/ml oleic acid plus 50 µg/ml cholesterol. * *p* < 0.05 considered as statistically significant

Oil Red O staining revealed that LDs were present in D-17 and to a lesser extent in COS4288 cells when cells were cultivated in monolayer under standard conditions (Fig. 2A, B). Treatment with oleic acid alone (Fig. 2C, D) or in combination with cholesterol dramatically increased the amount of lipid droplets in D-17 and COS4288 monolayers, whereas supplementation with cholesterol alone did not result in augmentation of lipid droplet formation. Treatment with ethanol (solvent control) did not have an effect on lipid droplets (data not shown).

The colorimetric quantification of the Oil Red O staining revealed a statistically significant increase of Oil Red O signal in oleic acid treated D-17 and COS4288 cells (Fig. 2E, F). A combination of oleic acid and cholesterol treatment resulted in a comparable enhancement of signal in both cell lines. In contrast, no relevant differences were measured in cholesterol alone treated cells. An influence of ethanol as solvent was excluded, as no differences between ethanol treated cells and cells grown under standard conditions were seen.

**Fig. 3:**
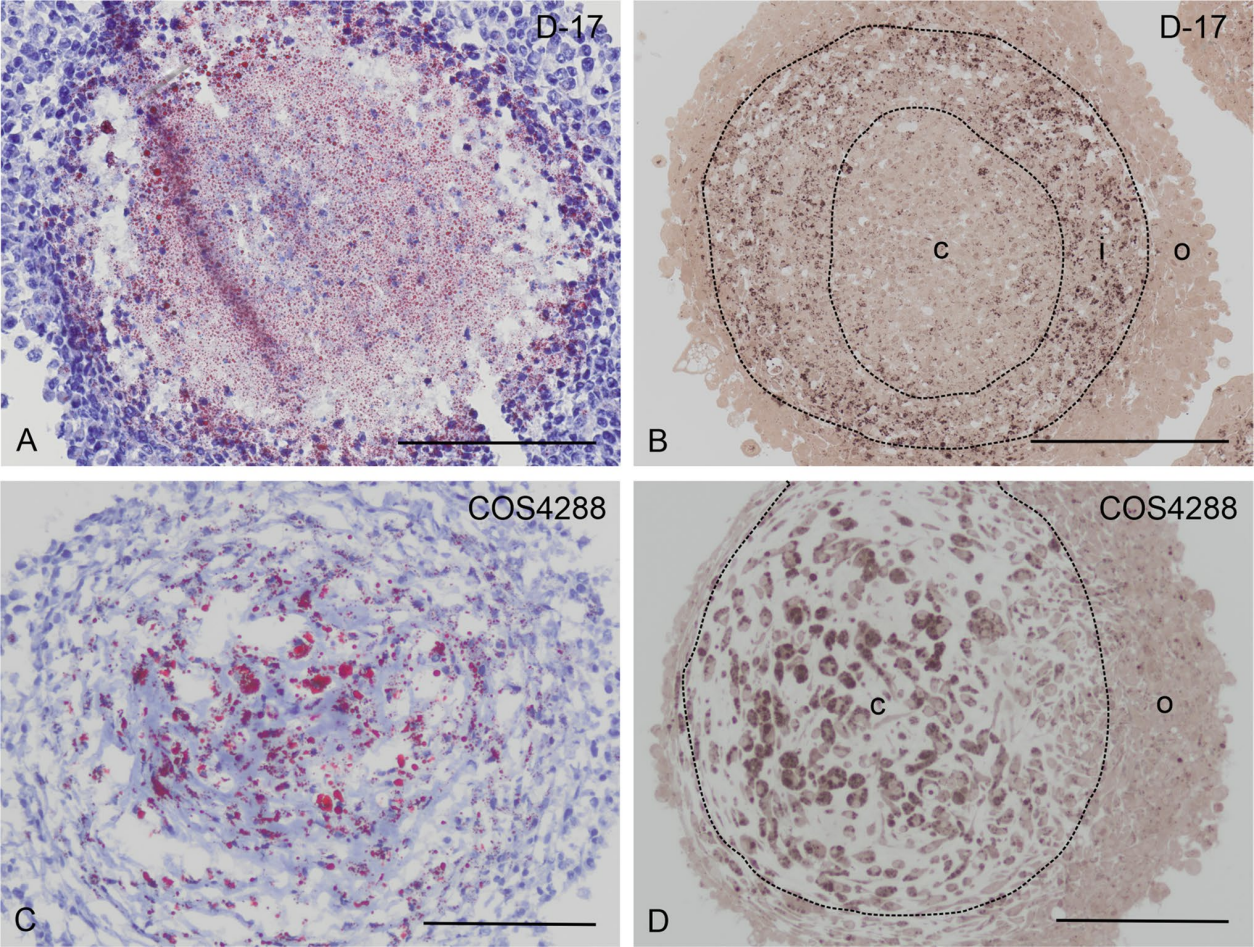
Lipid droplets detection in D-17 and COS4288 spheroids cultivated under standard conditions for 14 days. In D-17 spheroids, Oil Red O stained lipid droplets (red signal) were detected predominantly in the central and intermediate zone (A), whereas PPD-positive lipid droplets (brown signal) were localized in the intermediate zone (B). In COS4288 spheroids, majority of Oil Red O (C) and PPD-stained (D) lipid droplets was found in the central zone. Zones: c = central zone, i = intermediate zone, o = outer zone. Scale bar 200 µm

It is generally accepted that 3D tumor models mimic the *in vivo* situation better than cells grown in a standard monolayer culture (2D system). Therefore, we generated 3D spheroids from both cell lines (D-17, COS4288) and investigated the presence and distribution of lipid droplets in these microtumor models. We confirmed the presence of lipid droplets using PPD and Oil Red O staining in spheroids at all harvesting time points (7, 14 and 21 days of cultivation). However, LDs were distributed heterogeneously within the spheroids, showing a highly specific pattern within the respective cell lines´ spheroids. We were able to identify three zones within the D-17 spheroids (central, intermediate and outer zone) whereas COS4288 spheroids were characterized by only two clearly distinguishable zones (central and outer zone). Oil Red O staining in D-17 spheroids revealed LDs predominantly in the central and intermediate zone, whereas PPD-positive LDs were localized mostly in the intermediate zone (Fig. 3A, B). In contrast, the distribution pattern of Oil Red O and PPD-positive lipid droplets in COS4288 corresponded with the majority of lipid droplets located in the central zone (Fig. 3C, D). The distribution of LDs did not change with the cultivation time of the spheroids (data not shown).

### Immunfluorescence of PLINs on cell cultures

**Fig. 4:**
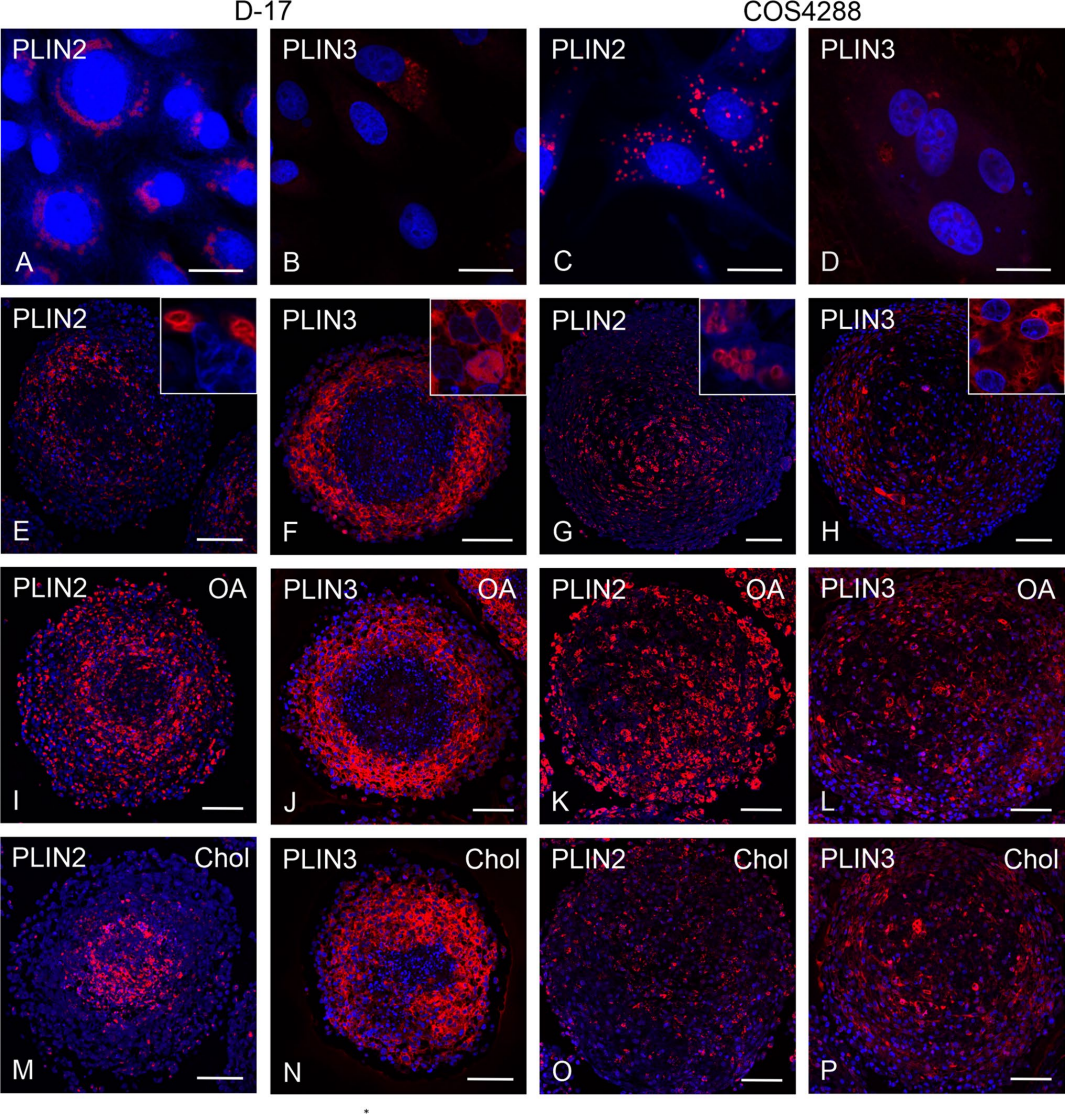
Immunofluorescence detection of PLIN2 and PLIN3 in D-17 and COS4288 cells cultivated under standard conditions and in spheroids after lipid supplementation. PLIN2 and PLIN3 expression was observed in monolayers of both D-17 (A, B) and COS4288 (C, D) cell lines. In D-17 spheroids, PLIN2 expression was high in the intermediate zone (E), whereas in COS4288 spheroids positive cells were mainly seen in the central zone (G). Zonal distribution of the PLIN3 signal was present in both cell lines with highest expression in the outer and intermediate zone in D-17 spheroids (F) and no clear zonal distribution in COS4288 spheroids (H). After stimulation with oleic acid (OA), PLIN2 staining occupied all spheroid zones (I, K) whereas after cholesterol (Chol) treatment PLIN2 labelling was predominant in the D-17 spheroid center (M). In COS4288 spheroids, the PLIN2 signal was distributed over all zones (O). PLIN3 staining pattern in D-17 spheroids was similar in spheroids grown under standard conditions and after lipid supplementation (F, J, N). In COS4288 spheroids, lipid stimulation revealed positive PLIN3 staining in all zones (L, P). Shown are spheroids cultivated for 14 days. PLIN2/PLIN3 (red), nuclei are counterstained with DAPI (blue). Scale bar 20 µm (A-D) and 100 µm (E-P)

Immunofluorescence for PLIN detection was applied on D-17 and COS4288 cells grown as monolayer (Fig. 4A-D) as well as in spheroids for 7, 14 (Fig. 4E-H), and 21 days. In line with our previous observations, PLIN1 signal was neither detected in D-17 nor in COS4288 cell cultures (not shown). PLIN2 expression was observed in monolayers in both cell lines. Similar to the PLIN2 staining of tumor tissue, signals corresponded to LD membranes (Fig. 4A, C). PLIN3 was detected in the cytoplasm of monolayers of both cell lines (Fig. 4B, D). Occasionally PLIN3 signal was observed in the nuclei in COS4288 cells.

In spheroids of both cell lines differences in zonal distribution of PLIN2 were seen. While in D-17 cells PLIN2 expression was strong in the intermediate zone but rather weak in the spheroid center (Fig. 4E), the most intense signal in COS4288 cells was detected in the central zone and only weak staining was seen in the outer zone (Fig. 4G).

In contrast to PLIN2, the PLIN3 signal tended to be stronger in the outer zone compared to the center in COS4288 spheroids but showed no clear zonal allocation (Fig. 4H). In D-17 spheroids, PLIN3 positive cells were seen predominantly in the outer and intermediate zone with almost no signal in the spheroid center (Fig. 4F). This general signal pattern was independent from the spheroid cultivation time. PLIN2 staining was more pronounced in D-17 and COS4288 spheroid cultures after OA supplementation and PLIN2 positive LDs occupied all spheroid zones (Fig. 4I, K). The distribution of PLIN3 was comparable in D-17 spheroids grown under standard condition and after lipid treatments (Fig. 4 F, J N). In lipid supplemented COS4288 spheroids, PLIN3 signal was spreading to the outer layers of the spheroids (Fig. 4 L, P). In Chol treated D-17 spheroid cultures, PLIN2 signal was predominant in the spheroid center (Fig. 4M), whereas in COS4288, PLIN2 marked LDs were sparse and regularly distributed within the whole spheroid.

### Western Blot analyses of PLINs

**Fig. 5:**
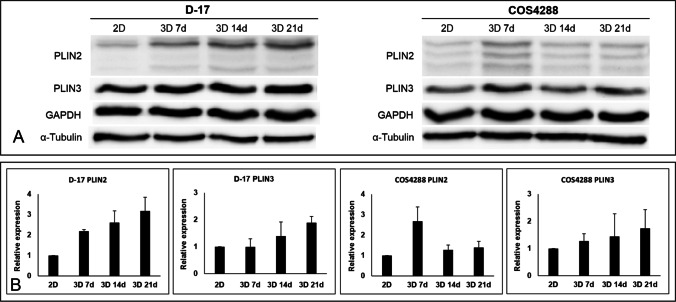
Western Blot analysis of PLIN2 and PLIN3 expression in D-17 and COS4288 cells cultivated under standard condition as a monolayer (2D) or spheroids (3D) for 7, 14, and 21 days. (A) Representative Western Blot. PLIN2 detection in COS4288 cells resulted constantly in the presence of three bands (approx. 45, 50, 52 kDa), whereas D-17 cells showed only one prominent band at approx. 52 kDa and two smaller very faint bands. (B) Semiquantitative analysis of PLIN2 and PLIN3 expression relative to GAPDH (PLIN2) or α-Tubulin (PLIN3), respectively. Higher levels of PLIN2 and PLIN3 proteins were detected in spheroids as compared to monolayer cells. Their expression levels increased with spheroid cultivation time except for PLIN2 in COS4288 cells. * *p* < 0.05 considered as statistically significant

The expression of PLIN1, PLIN2 and PLIN3 in monolayer and in spheroids cultivated under standard conditions was further investigated using semi-quantitative Western Blot (Fig. 5A, B). In line with our previous observations, PLIN1 was undetectable in both D-17 and COS4288 cells (not shown). For the other two PLIN proteins investigated, a trend towards increased protein levels was seen in spheroids as compared to cells grown in monolayer. Additionally, higher levels of PLIN2 and PLIN3 expression were observed in spheroids of both cell lines cultivated for 21 days as compared to spheroids cultivated for 7 days, with exception of PLIN2 in COS4288 cells peaking at 7 days (Fig. 5B). Nevertheless, none of the observed trends were statistically significant. Surprisingly, PLIN2 detection in COS4288 cells resulted constantly in the presence of three bands (approx. 52, 50, 45 kDa), whereas D-17 cells showed one prominent band at approx. 52 kDa and two smaller, very faint bands (Fig. 5A).

**Fig. 6:**
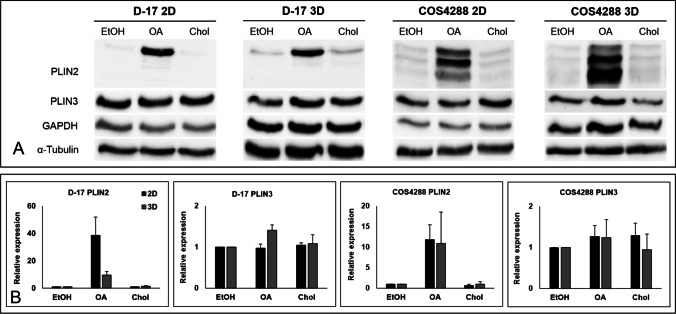
Western Blot analysis of PLIN2 and PLIN3 expression in D-17 and COS4288 cells cultivated as a monolayer (2D) or spheroids (3D) with supplementation of oleic acid (OA) and cholesterol (Chol). (A) Representative Western Blot. (B) Quantification of PLIN2 and PLIN3 expression relative to GAPDH (PLIN2) or α-Tubulin (PLIN3), respectively. Upon OA supplementation, PLIN2 expression was upregulated in D-17 as well as in COS4288 cells in both, monolayer and spheroid samples. Ethanol (EtOH) served as solvent control

While cholesterol supplementation did not have an effect on PLIN2 and PLIN3 protein amount, oleic acid treatment increased the protein levels of PLIN2 in monolayer and spheroid samples of D-17 (only the prominent band at 52 kDa) as well as COS4288 cells (all three bands, Fig. 6A, B).

### Quantitative real-time PCR for PLINs

**Fig. 7:**
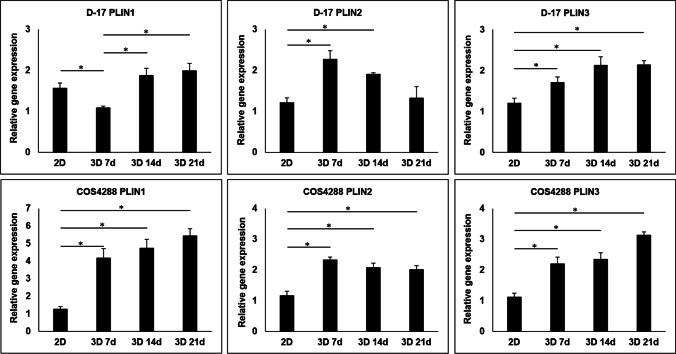
Relative PLIN1, PLIN2, and PLIN3 gene expression in D-17 and COS4288 cells cultivated without lipid supplementation as a monolayer (2D) or spheroids (3D) for 7, 14, and 21 days. With exception of PLIN2, a progressive increase in mRNA levels was observed with longer spheroid cultivation time. * *p* < 0.05 considered as statistically significant

A general trend towards higher mRNA levels of PLIN1, PLIN2, and PLIN3 was observed in D-17 and COS4288 spheroids as compared to cell monolayers, both cultured under standard conditions (Fig. 7). In D-17 cells, significant differences were seen in PLIN1 mRNA levels between spheroids cultivated for 7 days and all other time points. Significantly more PLIN2 mRNA transcript was detected in spheroids cultivated for 7 and 14 days as compared to cell monolayers, however the general trend was a decrease. In terms of PLIN3, D-17 cells showed significantly higher mRNA levels of PLIN3 in spheroids compared to cells cultivated as monolayers and a further increase with spheroid cultivation time (Fig. 7).

In COS4288 cells, a progressive increase in mRNA levels was observed with longer spheroid cultivation time in PLIN1 and PLIN3. In accordance with the data observed by Western Blot analysis, PLIN2 mRNA levels peaked in spheroids cultivated for 7 days, and after a small decrease, these levels stayed constant in spheroids cultivated for 14 and 21 days (Fig. 7).

### Cholesterol and triacylglyceride assay in osteosarcoma cells *in vitro*

To gain more information about the LD composition, we measured the cholesterol and triacylglyceride content in D-17 cells grown in a monolayer as well as in spheroids (cultivation time 7, 14, and 21 days) under standard conditions.

**Fig. 8:**
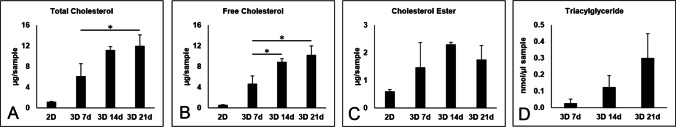
Quantification of cholesterol and triacylglyceride content in D-17 cells grown in a monolayer as well as in spheroids (cultivation time 7, 14, and 21 days) under standard conditions. The amount of total and free cholesterol as well as triacylglyceride amount in spheroids increased with prolonged cultivation time. * *p* < 0.05 considered as statistically significant

All forms of cholesterol were present in cells grown as a monolayer. In spheroids, the amount of total and free cholesterol significantly increased with prolonged cultivation time, whereas the changes in esterified cholesterol were only marginal (Fig. 8A-C). A statistical comparison between monolayers and 3D cell culture models in this assay is not possible due to the nature of sample-preparation and harvesting.

The amount of triacylglyceride was under the detection limit of the used kit in cells cultivated as a monolayer. The triacylglyceride amount increased with the spheroid cultivation time, however this trend was not statistically significant (Fig. 8D).

### Analyzes of lipid droplet size by transmission electron microscopy

After LD detection in 3 independent tumor tissues via electron microscopy, we further analyzed the size of LDs in the original tumor tissue of COS4288, as well as in D-17 and COS4288 cells grown under standard conditions as monolayers and as 3D spheroids (cultivation time 7, 14, and 21 days).

**Fig. 9:**
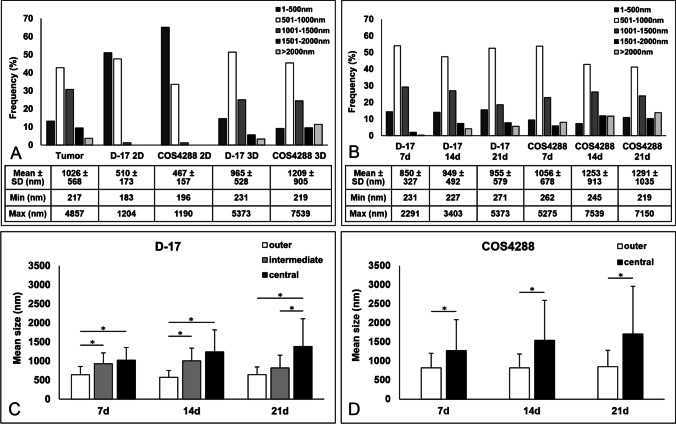
Size analysis of lipid droplets via electron microscopy. The frequency of LD sizes as well as the mean, the minimal and the maximal values of 3D cultured cells resembled the results obtained from tumor tissue whereas cells grown in a monolayer contained smaller lipid droplets (A). Cultivation time of spheroids did not change the overall frequency of LD sizes, but percentage of lipid droplets > 2000 nm as well as the max values increased over time (B). Analyzing the zonal distribution in D-17 (C) and COS4288 (D) spheroids, the mean lipid droplet size increased from outer layers towards center of the spheroids. * *p* < 0.05 considered as statistically significant

**Fig. 10:**
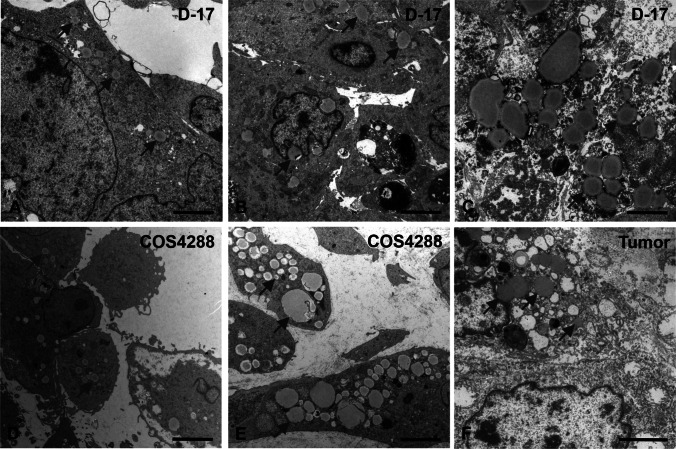
Electron microscopy microgaphs of lipid droplets (black arrows) in D-17 ((A) outer, (B) intermediate, (C) central zone) and COS4288 ((D) outer, (E) central zone) spheroids cultivated for 14 days under standard conditions. (F) canine osteosarcoma tumor tissue originating from COS4288 primary tumor. Scale bar 2500 nm

The frequency of LD sizes as well as the mean, the minimal and the maximal values of 3D cultured cells resembled the results obtained from tumor tissue (Figs. 9A and 10F). In contrast, cells grown in a monolayer contained smaller LDs. The average values of LD sizes were significantly smaller in monolayers compared to 3D spheroids of D-17 and COS4288 cells at all time points. Cultivation time of spheroids did not change the overall frequency of LD sizes, however, percentage of LDs > 2000 nm as well as the maximal values increased over time (Fig. 9B).

In accordance with paraffin-embedded samples, three zones (central, intermediate and outer zone) could be identified in D-17 spheroids, whereas in COS4288 only a central and an outer zone were seen. The mean sizes of LDs varied within these zones (Figs. 9C, D and 10A-E), With the LDs being significantly smaller in the outer zone in both cell lines at all harvesting time points. The LD size variation was the largest in the central area, mainly in spheroids cultivated for 21 days, whereas in the intermediate and/or outer zone the LD size was more homogeneous (Fig. 9C, D). The estimated amount of LDs observed in the central zone was superior to those observed in intermediate and/or outer zone.

## Discussion

Sixty-five years have passed since Novikoff identified the lipid nature of cytoplasmic inclusions in rat tumor cells for the first time (Novikoff 1957). Regarded as inert fat storage depots, the significance of these inclusions, later addressed as lipid droplets, was neglected for many years. Along with the increasing knowledge on the subcellular organization, molecular processes inside the cells as well as lipid biology and chemistry, LDs have progressively become more of a key player. It is now clear that they are not only involved in cellular energy metabolism, but also in cell signaling, homeostasis and different cellular processes associated with pre-metastatic niche formation, cancer cell survival, progression and aggressiveness (Cruz et al. 2020; Cortini et al. 2021; Koundouros and Poulogiannis 2020; Petan et al. 2018; Royo-Garcia et al. 2021; Shang et al. 2020).

In the current study, we have shown that LDs are present in a considerable amount in canine osteosarcoma tissue samples as well as in canine osteosarcoma cell lines. LDs were unevenly distributed in the tumor and in the 3D model, where more LDs were observed in the central and intermediate zone of the spheroids. This observed pattern in spheroids corresponds to the expected hypoxic regions in the central part of the spheroids (Lin and Chang 2008). Our observation is congruent with previous research, where LD accumulation has been observed in hypoxic/acidic regions (Koizume and Miyagi 2016; Cortini et al. 2021). In COS4288 spheroids, only two zones were clearly distinguishable. We speculate that the absence of a distinct intermediate zone in COS4288 spheroids is due to a better nutrient accessibility caused by a less compact arrangement of cells. Similarly, in the osteosarcoma tissue the uneven distribution probably reflects the cellular heterogeneity of the tumor and spatial distribution of areas with different oxygen supply as previously shown for other tumor types (Corre et al. 2020; Vaupel and Harrison 2004). LDs placement has been mapped in the necrotic tissue and in hypoxic tissue adjacent to necrosis in orthotopic rat C6 gliomas (Zoula et al. 2003) and in a human comedo-type ductal carcinoma *in situ* (Vidavsky et al. 2019).

Further investigations should focus on which types of cells in an OS are especially capable of LD formation, storage, and signaling as multiple players could be involved, including tumor cells, tumor surrounding/stroma cells along with macrophages, and giant cells.

Lipid droplet coating proteins were detected in tissue samples of canine osteosarcoma patients as well as in OS cell lines. In tissue samples, PLIN1, PLIN2 and PLIN3 were detected in varying distributions, however, PLIN1 was restricted to adipose tissue cells. This data corresponds to results obtained from our 3D cell culture models. PLIN2 and PLIN3 were detected in 3D culture models by means of immunofluorescent staining as well as Western Blot analysis, while all samples were negative for PLIN1. Despite the absence of PLIN1 at the protein level, its mRNA was detected in D-17 and COS4288 cells, irrespective of the model applied. This apparent contradiction is in line with the observations of others, as it is accepted protein expression does not always correlate with mRNA expression (Shirasaka et al. 2009).

Our histochemical and immunofluorescence analyses revealed that location of PLIN2 with respect to the subcellular staining pattern and the distribution pattern inside the spheroids resembles to a large extent the distribution of LDs observed with Oil Red O and PPD staining. Spatial distribution of PLIN2 found in D-17 and COS4288 3D model located to the hypoxic area in the middle of the spheroids is in accordance with previous observations, describing increased PLIN2 protein levels associated with necrotic regions of human breast carcinoma (Kuniyoshi et al. 2019). Moreover, high PLIN2 expression levels have been linked to several tumor types including human breast, colon, kidney and lung tumors (summarized in Cruz et al. 2020).

In contrast to PLIN2 expression, PLIN3 was observed predominantly in the marginal zone of the spheroids. In addition, occasional clear nuclear PLIN3 signals were observed. Similar PLIN3 nuclear signal in absence of PLIN2 has been described in hepatocyte derived cell lines, clearly associating this signal with nuclear lipid droplets (Ohsaki et al. 2016). The presence of nuclear lipid droplets has been proven in cells of different origin (Uzbekov and Roingeard 2013; Romanauska and Köhler 2018; Sołtysik et al. 2019), including the human osteosarcoma cell line U-2OS (Sołtysik et al. 2020) and should therefore not be neglected in further studies.

Based on the semiquantitative Western Blot data analyses, a trend towards cultivation time dependent increase of PLIN3 protein amount in spheroids was noticed in both cell lines, which correlates with the relative gene expression at the mRNA level. In D-17 cells, a clear and repeatable increase in PLIN2 protein expression level was demonstrated with prolonged spheroid cultivation time, whereas the mRNA levels peaked after 7 days of cultivation. Both protein and mRNA levels of PLIN2 in COS4288 cells reached their maximum at 7 days of cultivation. Interestingly, the Western Blot signals for PLIN2 protein differed substantially between the analyzed cell lines. Whereas D-17 cells revealed one prominent PLIN2 corresponding band, three distinctive and well-defined bands were present in COS4288 cells. A similar observation of multiple bands obtained after PLIN2 protein detection for well-differentiated human liposarcoma tissue samples has already been illustrated by Straub et al. (2019), however without particular explanation. Russel et al. (2008) described a N-terminally truncated form of PLIN2 in mouse mammary gland arising from the alternative translation initiation site in the PLIN2 gene. Consistent with the infrequent literature data available for PLIN2 size differences in species other than dog, we speculate that the presence of different isoforms, splice variants, posttranslational modifications as well as aberrant translation initiation or termination might be a putative source of this observation. Thus, to elucidate the origin of the multiple bands detected by the PLIN2 antibody further investigations are required.

As previously described, LDs consist predominantly of triacylglycerols and cholesteryl esters located in the LD core, surrounded by a monolayer of phospholipids and various LD-associated proteins (Cruz et al. 2020). In our 3D culture system, cholesterols (total cholesterol and free cholesterol) as well as triacylglycerols could be detected, with amounts increasing with culture time. Nevertheless, this data has to be interpreted with care, as we cannot exclude that the observed increase in measured parameters was influenced by the rising cell count caused by physiological cell division reflecting the different cultivation times. Further insight into the distribution of cholesterols and triacylglycerols in tumor tissue sections would be desirable; however, *in situ* this is only possible on lipid preserving cryosections, which is challenging in a calcified tissue such as bone-tumor.

Our data clearly showed a stimulating effect of oleic acid, used at the concentration previously described for *in vitro* studies (Fan et al. 2013). This was true for both, LDs and PLIN2 expression in both osteosarcoma cell lines. Oleic acid induced lipid accumulation has also been observed in several other cancer cell lines, including those arising from cervical, breast, and hepatocellular carcinoma (Guštin et al. 2017; Pucer et al. 2013; Giulitti et al. 2021). In contrast to the stimulating capacity of OA, no effect was found for supplemented cholesterol. Our results revealed that both osteosarcoma cell lines were susceptible for externally provided lipid oleic acid but not cholesterol. Data about lipids in OS are scarce, even for human OS. However, one study has shown a potential role of cholesterol in canine OS biology as elevated serum levels have been detected in tumor-bearing dogs (Leeper et al. 2017). Clearly, more studies on lipids and LDs as well as their relationship in OS are warranted.

The described size of LDs vary widely between the cells or even within the same cell ranging from a few dozen nanometers to hundreds of micrometers (Yang et al. 2012). Based on our electron microscopic analysis, the LD size range was between few hundred to several thousand nanometer in the canine OS. Only the 3D cell culture tumor model revealed a size range of LDs and frequency of LD sizes similar to the original tumor tissue. This once more indicates the importance of 3D *in vitro* systems to mimic the *in vivo* tumor microenvironment and shows the weakness of monolayer approaches. The complex 3D conditions to which these cells are exposed, represent a realistic tissue architecture and therefore are more physiologically relevant (Pinto et al. 2020; El Atat et al. 2022; Rossi et al. 2022). Except of the maximum size of the LDs, which was detected after 14 days of spheroid cultivation, we only found few differences between the different cultivation durations. All analyzed parameters were already detectable after 7 days of cultivation. We therefore suggest that spheroids cultured for 7 days represent a valuable model to study LDs in canine OS in the future.

In conclusion, we have proven a significant amount of LDs in canine OS tissue samples as well as in the canine OS cell models *in vitro*. Lipid droplet coating proteins of the perilipin family (PLIN2, PLIN3) were detected in both, tumor samples and canine osteosarcoma cell lines D-17 and COS4288 as well. Size range of LDs, frequency of LD sizes, and heterogeneous LD distribution in 3D spheroid were similar to the situation in the corresponding naturally occurring canine OS from the same patient. Thus, 3D spheroids are a relevant *in vitro* model for further studies on canine OS focusing on lipid droplet biology and function (e.g. influence on chemotherapy resistance). A large-scale study for LDs and PLINs, covering the wide range of patient derived OS subtypes, grades, stages and clinical outcomes would be desirable to elucidate their mechanisms and functions in tumor development and progression.

## Supplementary Information

Below is the link to the electronic supplementary material.

Supplementary file1 (DOCX 28 KB)

Supplementary file2 (JPG 3666 KB)

## Data Availability

The datasets generated during and/or analyzed during the current study are available from the corresponding author on reasonable request.
