# Supplementary material for: Lipid droplets and perilipins in canine osteosarcoma. Investigations on tumor tissue, 2D and 3D cell culture models
Source: Vet Res Commun. 2022 Jul 14;46(4):1175–93. doi: 10.1007/s11259-022-09975-8 (PMC9684256; doi:10.1007/s11259-022-09975-8)
Supplement: Supplementary file 1 — Supplementary file1 (DOCX 28 KB) [file 11259_2022_9975_MOESM1_ESM.docx]

**Characterization of canine osteosarcoma cell line COS4288**

**Materials and Methods**

Continuously growing COS4288 cells were cultivated for at least 30 passages before entering the characterization process. The morphology of the cells was documented using EVOS FL Auto microscope (Life Technologies, Carlsbad, CA, USA).

**Soft agar colony formation assay**

The soft agar colony formation assay was performed as described by Borowicz, using 2x10^3^ cells/cm^2^ of the surface area (Borowicz et al. 2014). Briefly, wells of the 6-well plate (Sarstedt, Nümbrecht, Germany) were first covered with 0.5% agar in DMEM high glucose medium (both Sigma-Aldrich, St. Louis, MO, USA) and overlaid afterwards with 0.3% agar/DMEM containing the cell suspension. After solidification (30min at room temperature), 200µl of cultivation medium was added to each well. The ability of COS4288 cells to form colonies in soft agar was monitored after 21 days of cultivation using light microscopy (Zeiss Observer Z1, Oberkochen, Germany).

**Immunofluorescence and immunohistochemical analysis**

Cells were grown on a 4-well glass chamber slide (Lab-Tek II Chamber Slide System, ThermoFisher Scientific, Waltham, MA, USA).

For detection of cytokeratin and vimentin, fixation (10min) was done with ice-cold methanol (Carl Roth, Karlsruhe, Germany). For cytokeratin detection, endogenous peroxidases were blocked with 3% H_2_O_2_ for 60min at room temperature followed by antigen retrieval in Tris-EDTA pH 9. Unspecific binding of the antibody was blocked with 10% normal goat serum in PBS (both Sigma) for 60min at room temperature. A cocktail of monoclonal mouse anti-cytokeratin HMW [clone AE3] (cat.# BSB5445, BioSB, Santa Barbara, CA, USA; dilution 1:500) and monoclonal mouse anti-cytokeratin LMW [clone AE1] (cat.# 301M-14, Cell Marque, Sigma; dilution 1:500) was applied overnight at 4°C. Antibody binding was detected using BrightVision Poly-HRP anti-mouse (cat.# DPVM-HRP, Immunologic, Duiven, Netherlands, ready-to-use) secondary system. Signal of the secondary HRP labelled antibody was visualized using Invitrogen™ Alexa Fluor™ 488 Tyramide-Reagent (Invitrogen, Waltham, MA, USA).

For vimentin detection, antigen retrieval was performed in citrate buffer pH 6. Unspecific binding of the antibody was blocked by sample incubation in 1.5% normal goat serum in PBS (both Sigma; 60min at room temperature). Mouse monoclonal anti-vimentin [clone V9] antibody (cat.# M0725, Dako Agilent Technologies, Santa Clara, CA, USA, dilution 1:100) was applied over night at 4°C and followed by incubation with goat anti-mouse IgG (H+L) highly cross adsorbed AlexaFluor488-conjugated secondary antibody (cat.# A11029, Invitrogen dilution 1:100) for 60min at room temperature.

For both stainings (cytokeratin and vimentin), nuclei were counterstained using DAPI (4′,6-diamidino-2-phenylindole, Sigma Aldrich) and mounted with coverslip and Aqua Poly Mount^TM^ (Polysciences, Warrington, PA, USA). Images were taken using a confocal laser scanning microscope (Zeiss CLSM 880 Airyscan).

For immunohistochemical detection of alkaline phosphatase (ALPL) and karyopherin α2 (KPNA2), cells were fixed in 4% neutral buffered formaldehyde for 10min at room temperature. Cell permeabilization was performed by incubation in 0.2% TritonX-100 (Merck, Darmstadt, Germany) in PBS for 15min at 4°C. Endogenous peroxidases were blocked with 0.6% H_2_O_2_ in methanol for 15min, followed by an antigen retrieval for 30min in 0.01M citrate buffer pH 6. After protein block in 1.5% normal goat serum in PBS, samples were incubated with the following primary antibodies overnight at 4°C: polyclonal rabbit anti-alkaline phosphatase (ALPL) antibody (cat.# GTX100817, Genetex, Irvine, CA, USA; dilution 1:100) and polyclonal rabbit anti KPNA2 antibody (cat.# HPA041270, Sigma Prestige, dilution 1:500). Signals were detected with the BrightVision Poly-HRP-anti-rabbit system (ImmunoLogic) using DAB-solution (Quanto, Richard Allan Scientific, Kalamazoo, MI, USA) as a chromogene. Finally, samples were counterstained with hematoxylin (Epredia, Richard Allan Scientific) and mounted with DPX medium (Fluka, Buchs, Switzerland). Evaluation of the samples was performed using light microscopy (Olympus BX53, Shinjuku, Japan).

**Results and Discussion**

We successfully isolated uniform, deltoid-shaped cells (Suppl. Fig. 1A) from a clinical sample of osteoblastic osteosarcoma and cultured them continuously for four months (30 passages) before we considered these cells to be a cell line (COS4288) and subjected them to further analyses with the aim to prove their osteosarcoma origin. Next to these analyses, COS4288 cells were continually kept in culture (for more than six months) and reached passage number over 50. In terms of this observation we presume that COS4288 cells derived from tumor. To further support this assumption, we investigated the anchorage-independent growth capability of COS4288 cells using the soft agar colony formation assay. In our experiments, COS4288 formed multicellular colonies in soft agar within 21 days (Suppl. Fig. 1B).

Anchorage-independent cell growth in soft agar is a well-established *in vitro* laboratory method, which serves, along with capability of unlimited cell division, as a hallmark of malignant transformation of cells (Hamburger et al. 1978, Raimondi et al. 2022). These results supported our presumption that the COS4288 cell line derived from OS tumor.

Osteosarcomas are mesenchymal tumors. In order to prove mesenchymal and to exclude epithelial origin of COS4288 cells, immunofluorescence stainings for the mesenchymal marker vimentin and epithelial marker cytokeratin were performed. As expected, cells presented vimentin-positive intermediate filaments (Suppl. Fig. 1C) whereas cytokeratin signal was absent (Suppl. Fig. 1D), confirming mesenchymal origin of COS4288 cells. Obtained data are in agreement with those previously reported for other canine osteosarcoma cell lines (Meyer and Walter 2016, Modesto et al. 2020).

As the only connective tissue producing ALPL in dogs is bone, it can be used to differentiate canine osteosarcoma from other vimentin-positive tumors (Barger et al. 2005). Indeed, several authors reported ALPL expression in osteosarcoma cell lines of both human (Laschi et al. 2015) and canine origin (Meyer and Walter 2016, Wilson-Robles 2019). Therefore positive ALPL immunohistochemical staining of COS4288 cells (Suppl. Fig. 1E) underlines the assumption of osteosarcoma origin of the respective cell line.

Overexpressed KPNA2 was observed in different tumors and it was linked with poor outcome in patients (Han et al. 2020). Recent study has shown its applicability for differentiation between osteosarcomas and other bone tumors, with expression across various types of osteosarcoma (Jiang et al. 2020). In COS4288 cells, KPNA2 protein was detected in the nuclei and in cytoplasm (Suppl. Fig. 1F), which is in agreement with the staining pattern previously observed in osteosarcoma (Jiang et al. 2020), and therefore provides further evidence for osteosarcoma origin of COS4288 cell line.

**Figure legend**

**Supplementary Figure 1** COS4288 cell characteristics. (A) Representative picture of COS4288 monolayer revealing deltoid-shaped cell morphology. (B) Multicellular colony formed in soft agar. (C) Vimentin immunofluorescence staining (green) indicating mesenchymal origin of COS4288 cells. Nuclei were counterstained with DAPI (blue). (D) Cytokeratin immunofluorescence staining was negative in COS4288 cells, excluding epithelial origin of the isolated cells. Insert shows cytokeratin signal (green) in canine endometrium used as positive control. Nuclei were counterstained with DAPI (blue). (E) Immunohistochemical detection of ALPL (brown) of cultivated COS4288 cells. Sample was counterstained with hematoxylin (blue). (F) KPNA2 immunohistochemical detection (brown) in nuclei and cytoplasma of COS4288 cells. Sample was counterstained with hematoxylin (blue). Scale bars (A) 500µm, (B‑F) 50µm.

**References**

Barger A, Graca R, Bailey K, Messick J, De Lorimer LP, Fan T, Hoffmann W (2005) Use of alkaline phosphatase staining to differentiate canine osteosarcoma from other vimentin-positive tumors. Vet Pathol 42:161-165. [doi: 10.1354/vp.42-2-161](https://doi.org/10.1354%2Fvp.42-2-161)

Borowicz S, Van Scoyk M, Avasarala S, Rathinam MKK, Tauler J, Bikkavilli RK, Winn RA (2014) The soft agar colony formation assay. J Vis Exp 92, e51998. doi: 10.3791/51998

Hamburger AW, Salmon SE, Kim MB, Trent JM, Soehnien BJ, Alberts DS, Schmidt HJ (1978) Direct cloning of human ovarian carcinoma cells in agar. Cancer Res 38:3438-3444.

Han Y, Wang X (2020) The emerging roles of KPNA2 in cancer. Life Sciences 241:117140. doi: 10.1016/j.lfs.2019.117140

Jiang L, Liu J, Wei Q, Wang Y (2020) KPNA2 expression is a potential marker for differential diagnosis between osteosarcomas and other malignant bone tumor mimics. Diagnostic Pathology 15:135. doi: 10.1186/s13000-020-01051-6

Lashi M, Bernardini G, Geminiani M, Ghezzi L, Amato L, Braconi D, Millucci L, Frediani B, Spreafico A, Franchi A, Campanacci D, Capanna R, Santucci A (2015) Establishment of four new human primary cell cultures from chemo-naïve Italian osteosarcoma patients. J Cell Physiol 230:2718-2727. doi: 10.1002/jcp/24996

Meyer FRL, Walter I (2016) Establishment and characterization of new canine and feline osteosarcoma primary cell lines. Vet Sci 3:9. doi: 10.3390/vetsci3020009

Modesto P, Fernandez JLC, Martini I, Zoccola R, Pugliano MC, De Ciucis CG, Goria M, Ferrari A, Razzuoli E (2020) Characterization of D-17 canine osteosarcoma cell line and evaluation its ability to response to infective stressor used as alternative anticancer therapy. Animals 10:1981. doi: 10.3390/ani10111981

Raimondi L, Gallo A, Cuscino N, De Luca A, Costa V, Carina V, Bellavia D, Bulati M, Alessandro R, Fini M, Conaldi PG, Giavaresi G (2022) Potential anti-metastatic role of the novel miR-CT3 in tumor angiogenesis and osteosarcoma invasion. Int J Mol Sci 23:705. doi: 10.3390/ijms23020705

Wilson-Robles H, Franks K, Pool R, Miller T (2019) Characterization of five newly derived canine osteosarcoma cell lines. BMC Vet Res 15:357. doi: 10.1186/s12917-019-2099-y

.
