# Supplementary figures and images for: Lipid droplets and perilipins in canine osteosarcoma. Investigations on tumor tissue, 2D and 3D cell culture models
Source: Vet Res Commun. 2022 Jul 14;46(4):1175–93. doi: 10.1007/s11259-022-09975-8 (PMC9684256; doi:10.1007/s11259-022-09975-8)

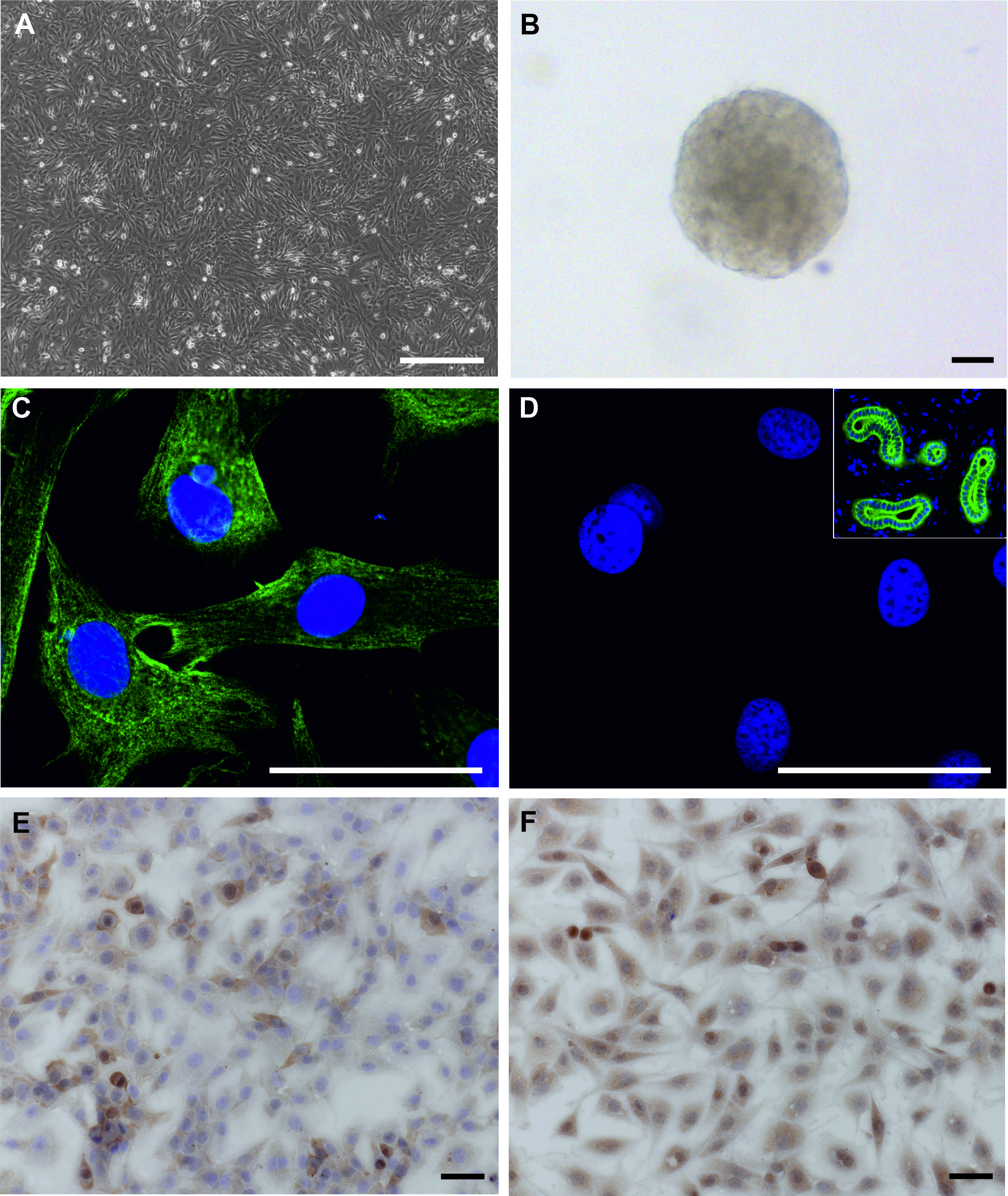

Supplement: Supplementary file 2 — Supplementary file2 (JPG 3666 KB) [file 11259_2022_9975_MOESM2_ESM.jpg]
